# Screening and Characterization of Antioxidant Film Applicable to Walnut Kernels from *Juglans sigillata*

**DOI:** 10.3390/foods13091313

**Published:** 2024-04-25

**Authors:** Ping Li, Yujia Zhang, Changwei Cao, Yaxi Luo, Huan Kan, Yun Liu

**Affiliations:** 1 Key Laboratory of Forest Resources Conservation and Utilization in the Southwest Mountains of China Ministry of Education, Southwest Forestry University, Kunming 650224, China; 15085852406@163.com (P.L.); a18111417100@163.com (Y.Z.); ccwylf1111@163.com (C.C.); kanhuan@swfu.edu.cn (H.K.); 2 Faculty of Human Nutrition Science, University of Manitoba, 66 Chancellors Cir, Winnipeg, MB R3T 2N2, Canada; lyx001013@outlook.com

**Keywords:** walnut kernel, *Juglans sigillata*, natural antioxidants, film, preservation

## Abstract

Walnuts play a positive role in human health due to their large amounts of unsaturated fatty acids, whereas lipid oxidation can easily occur during storage. Herein, three natural antioxidants (epicatechin, sesamol, and myricetin) were added to the composite film cross-linked with chitosan and soy protein peptide, and the antioxidant film appropriate for the preservation of walnut kernels from *Juglans sigillata* was screened to improve the storage quality of walnuts. The results showed that three antioxidant films could all enhance the storage performance of walnut kernels, with sesamol being the best. The characterization of antioxidant film cross-linked with chitosan and soy protein peptide containing sesamol (C/S-ses film) revealed that the composite film improved the slow release and stability of sesamol; in addition, the presence of sesamol could effectively reduce the light transmittance and water vapor permeability of the composite film, together with significantly enhancing the antioxidant and antimicrobial activities, resulting in an effective prolongation of the storage period of walnut kernels. These findings indicated that C/S-ses possess excellent potential for retarding the oxidative rancidity of unsaturated fatty acids and will provide an effective strategy for the preservation of walnut kernels.

## 1. Introduction

Among the kernels from the four major nuts (hazelnut, walnut, almond, and cashew), walnut kernels are rich in protein and lipid, as well as small amounts of vitamin E, minerals, and polyphenols, of which the oil content is higher than 60% [1,2]. Due to the presence of these nutrients, walnuts are often recognized as a brain-healthy food that improves brain memory [3] and has a positive role in preventing aging and age-related diseases [4]. Studies have found that unsaturated fatty acids in walnut kernels account for more than 90% of the total oils and fats, especially, the proportion of linolenic acid, which is significantly higher than other plants [5,6], with beneficial effects on human health through the regulation of blood lipid levels [7,8]. However, the unsaturated fatty acids in walnut kernels are prone to oxidative rancidity with the extension of storage time, resulting in a reduction of sensory properties such as the production of unpleasant odors, bitter tastes, and even sour tastes that are difficult for consumers to accept [9,10,11].

Various treatments have been used to extend the shelf life of walnuts, including low-temperature, gas conditioning, and ultraviolet irradiation [12], which are energy-intensive and costly. Recently, edible packaging films prepared from natural biopolymers like chitosan, alginate, and chitin have emerged as a research hotspot [13,14], which can preserve food while still reducing the production of plastic products [15,16]. In addition, to solve the shortcomings of single biomass films like poor mechanical properties, weak water and oxygen barrier capabilities, and bacterial inhibition [17], researchers have devoted themselves to the development of composite films with good performances and certain bioactivities, such as chitosan antimicrobial coatings, composite protein membranes, and smart response films [18,19,20]. Soy protein peptide is a purely natural nutrient extracted from soybeans with high nutritional value as well as important functional properties like good solubility, emulsification capacity, and antioxidant ability [21,22]. More than 70% of soy protein peptides are small-molecule peptides composed of 2–3 amino acids, which are easy to be absorbed by humans, improving the body’s immunity, reducing blood lipids, and inhibiting cholesterol [23]. Research has revealed that small-molecule peptides could be inserted into the polymer chains, thereby increasing the free volume of the film-forming matrix molecules and molecular mobility [24].

In recent years, nanoparticles, polyphenols, and flavonoids have been commonly used to modify films to improve tensile strength and water vapor resistance [25] and to prevent lipid peroxidation [26,27], which could facilitate the maintenance of organoleptic properties and prolong the shelf life of food [28,29]. Natural phenolic antioxidants like myricetin, epicatechin, and sesamol are widely found in plants and possess strong resistance to lipid peroxidation. Guitard et al. [30] have compared 22 natural polyphenols with 7 synthetic antioxidants in protecting omega (ω)-3 oils from autoxidation and found that myricetin could reduce the oxidation of ω-3 oils more effectively than alpha-tocopherol and synthetic antioxidants. Wang et al. [20] found that the chitosan-grafted epicatechin antioxidant film exhibited good impermeability to corn oil and could effectively delay the increase in thiobarbituric acid reactants and peroxide levels during oil storage. In addition, the study revealed that sesamol could provide better protection against oxidation for fish oils compared to commercial rosemary extracts at 30 and 50 °C [31].

With the objective of delaying lipid peroxidation of walnut kernels from *Juglans sigillata*, in the present study, chitosan was mixed with soy protein peptide to enhance the network structure, and different antioxidants (epicatechin, sesamol, and myricetin) were additionally added for preparing preservation films for walnut kernels. Subsequently, the antioxidant films were screened using the preservation effect as an indicator and characterized. This investigation will facilitate the application of antioxidant ingredients in food packaging.

## 2. Materials and Methods

### 2.1. Materials

Myricetin (98%), sesamol (99.9%), epicatechin (98%), chitosan (deacetylation degree ≥ 95%, a viscosity of 100–200 Pa·s), trichloroacetic acid, and thiobarbituric acid were acquired from Shanghai Adamasi Reagents Co., Ltd., (Shanghai, China). Soy protein peptide was obtained from Hubei Reborn Biotech Co., Ltd., (Jingzhou, China). Glycerol was obtained from Chengdu Cologne Chemical Co., Ltd., (Chengdu, China). Citric acid was obtained from Shanghai Aladdin Biochemical Technology Co., Ltd., (Shanghai, China) Calcium chloride anhydrous was obtained from Guangdong Guanghua Technology Co., Ltd., (Shantou, China). Benzene was purchased from Shanghai Macklin Biochemical Co., Ltd., (Shanghai, China). Walnuts were purchased from the Yongping County Bicheng Walnut Planting Farmers’ Specialized Cooperative. Other chemicals were of analytical grade.

### 2.2. Preparation of Film Fluid and Application to Walnut Kernels

Chitosan (1.6 g) was dispersed with soy protein peptide (0.4 g) into 100 mL of citric acid aqueous solution (4%), followed by the addition of 0.3% (*w*/*v*) antioxidant and 1% (*w*/*v*) glycerol. The above solutions were mixed in a magnetic stirrer at 50 °C for 3 h. Subsequently, chitosan solution (C), composite solution of chitosan and soy protein peptide (C/S), composite solution of chitosan and soy protein peptide containing epicatechin (C/S-epi), composite solution of chitosan and soy protein peptide containing sesamol (C/S-ses), composite solution of chitosan, and soy protein peptide containing myricetin (C/S-myr) were prepared.

Walnut kernels were separately poured into these five solutions, stirred rapidly for 1 min, and then fished out for drying at room temperature to obtain the coating of surface walnut kernels originating from the film solution (Figure S1). Subsequently, the walnut kernels covered with the coating were placed into self-sealing bags (120 g/per) and exposed to an electrothermal constant temperature blast drying oven (DHG-9240A, Qixin Scientific Instrument Co., Ltd., Shanghai, China) with accelerated oxidation for 24 d (40 °C). Indicators of walnut kernel oxidation were assessed as described every 4 d. Walnut oil was prepared using an expeller (K38, Yingshang Licheng Instrument Equipment Co., Ltd., Dongguan, China).

#### 2.2.1. Peroxide Value (PV)

The PV of walnut oil was determined using the Chinese standard of GB 5009.227-2016 [32].

#### 2.2.2. Acid Value (AV)

The AV of walnut oil was determined using the Chinese standard of GB 5009.229-2016 [33].

#### 2.2.3. Carbonyl Value (CV)

The CV of walnut oil was determined using the Chinese standard of GB 5009.230-2016 [34].

#### 2.2.4. Malonic Dialdehyde (MDA)

The MDA was determined according to the method of Sharma and Sharma [35] with minor modifications. Walnut kernel (1 g) was ground with 5.0 mL of trichloroacetic acid and centrifuged for 20 min. The above solution was boiled with 2.0 mL of thiobarbituric acid for 20 min and centrifuged to obtain the supernatant. The absorbance of the supernatant was determined at 450, 532, and 600 nm. The MDA was calculated as presented in Equation (1) as follows:

(1) $$\mathrm{MDA}\;(\mu \mathrm{mol}/g)=\frac{C\;\times \;V}{V_{s}\;\times \;m\;\times \;1000}$$

where *C* is the concentration of MDA in the reaction solution (μmol/L), *V* is the total volume of sample solution (mL), *V*_S_ is the volume of measurement sample (mL), and *m* is the weight of the sample (g).

#### 2.2.5. Conjugated Diene Value (K_232_) and Conjugated Triene Value (K_268_)

The K_232_ and K_268_ of walnut oil were determined using the Chinese standard of GB/T 22500-2008/ISO3656:2002 [36].

#### 2.2.6. Browning Degree

The browning degree was determined according to the method of Jiang [37], with minor modifications. Walnut kernel powder (1 g) was mixed with 20 mL of phosphate buffer solution (pH 6.8) and stirred for 15 min, followed by centrifugation at 8000 r/min for 10 min. The absorbance of the supernatant was measured at 450 nm.

#### 2.2.7. Moisture Content (MC)

The MC of walnut kernels was determined using the Chinese standard of GB 5009.3-2016 [38].

### 2.3. Preparation of Film

Air bubbles in the film solution (35 mL) were removed by sonication. The solution was poured into petri dishes with a diameter of 90 mm and dried in an oven (50 °C) to form a film. The film was then removed and placed in a desiccator with a relative humidity (RH) of 58% for 48 h before testing.

#### 2.3.1. Mechanical Properties and Thickness

The film was cut into 5 × 1.5 cm pieces and tested for tensile strength (TS) and elongation at break (EB) using a general-purpose mechanical testing machine (JHY-5000, Jinheyuan Technology Co., Ltd., Xiamen, China). The sample was stretched at a uniform speed (50 mm/min) until breakage. Film thickness was determined with an electronic digital caliper from at least five random locations. TS and EB were tested at least three times and calculated as shown in Equations (2) and (3) as follows:

(2) $$\mathrm{TS}\;(\mathrm{MPa})=\frac{E}{A\;\times \;B}$$

(3) $$\mathrm{EB}\;(\%)=\frac{L_{1}\;-\;L_{2}}{L_{2}}\;\times \;100\%$$

where *E* is the maximum load of the film at fracture (N), *A* is the film width (mm), *B* is the film thickness (mm), *L*_1_ is the film length at fracture (mm), and *L*_2_ is the initial film length (mm).

#### 2.3.2. Moisture Content and Water Solubility of Film

Moisture content of film and water solubility were determined according to the method of Jiang et al. [39], with minor modifications. The measurement of moisture content was that a 3 × 3 cm film strip was weighed and dried in an oven at 105 °C for 24 h. Water solubility was determined by drying the film (3 × 3 cm) for 24 h (45 °C), and the weights were recorded. The sample was then soaked in 30 mL of distilled water for 24 h and dried. The moisture content and water solubility of the sample were calculated as sown in Equations (4) and (5) as follows:

(4) $$\mathrm{Moisture}\;\mathrm{content}\;(\%)=\frac{m_{1}\;-\;m_{0}}{m_{1}}\;\times \;100\%$$

(5) $$\mathrm{Water}\;\mathrm{solubility}\;(\%)=\frac{m_{2}\;-\;{\;m}_{1}}{m_{2}}\;\times \;100\%$$

where *m*_1_ is the initial weight of the film (g), *m*_0_ is the weight of the film after drying (g), and *m*_2_ is the weight of the dried film after soaking (g).

#### 2.3.3. Water Vapor Permeability (WVP) and Oxygen Permeability (OP)

WVP was determined according to the method of Zhang et al. [40], with minor modifications. A centrifuge tube (10 mL) containing 3 g of anhydrous calcium chloride was sealed with film and placed in an enclosed container at 25 °C (75% of RH) for 48 h. Each experiment was repeated three times. In addition, OP was measured by replacing anhydrous calcium chloride with a deoxidizer. The WVP and OP of the sample were calculated as shown in Equation (6) as follows:

(6) $$\mathrm{WVP}/\mathrm{OP}\;(\frac{g\cdot \mathrm{mm}}{m^{2}\cdot h\cdot \mathrm{kPa}})=\frac{∆W\;\times \;d}{t\;\times \;A\;\times \;∆p}$$

where Δ*W* is the increment of the centrifuge tube (g), *d* is the thickness of the film (mm), *t* is the time interval (h), *A* is the area of the film (m^2^), and Δ*p* is the difference in water vapor pressure between the two sides of the film (kPa).

#### 2.3.4. Appearance and Color

The color of the film was measured using a colorimeter (SC-80, Beijing Kangguang Instrument Co., Beijing, China). For color parameters, *L* indicates lightness, *a* indicates redness/greenness, and *b* denotes yellowness/blueness. The white plate was chosen as a standard (*L*_0_, *a*_0_, and *b*_0_). The total color difference (Δ*E*) was calculated as shown in Equation (7) as follows:

(7) $$\Delta E=\sqrt{{\left(L^{*}\right)}^{2}+{\left(a^{*}\right)}^{2}+{\left(b^{*}\right)}^{2}}$$

where L* = *L* − *L*_0_, a* = *a* − *a*_0_, and b* = *b* − *b*_0_.

#### 2.3.5. Opacity

Opacity was determined according to the method of Park and Zhao [41], with minor modifications. The film was cut into 2 × 4 cm pieces and placed on one side of the cuvette to be measured at 600 nm. The opacity was calculated as shown in Equation (8) as follows:

(8) $$\mathrm{Opacity}\;(A_{600}/\mathrm{mm})=\frac{A_{600}}{L}$$

where *A*_600_ is the absorbance of the film at 600 nm and *L* is the film thickness (mm).

#### 2.3.6. Scanning Electron Microscope (SEM)

The microstructure of the film was observed by SEM (Regulus 8100, Hitachi Scientific Instruments Co., Ltd., Beijing, China). Briefly, the film was applied to conductive adhesive tape for gold plating. The test conditions were a gold spraying time of 60 s and an accelerating voltage of 3.0 kV.

#### 2.3.7. Fourier-Transform Infrared (FTIR) Spectroscopy

The molecular structure of the film polymer was examined via FTIR spectrometer (Nicolet iS50, Yu Hong Industry Co., Ltd., Shanghai, China) with the following acquisition parameters: scanning wavelength range of 4000–400 cm^−1^, spectral resolution of 4 cm^−1^, and 32 scans.

#### 2.3.8. Differential Scanning Calorimetry (DSC)

The thermal property of the film was evaluated by a differential scanning calorimeter (Mettler DSC3, Mettler Toledo., Zurich, Switzerland). The sample was heated from 30 °C to 150 °C at a rate of 10 °C/min.

#### 2.3.9. Antimicrobial Activity

Antimicrobial activity was determined according to the method by Ardjoum et al. [42], with slight modifications. Briefly, a piece of filter paper with a diameter of 6 mm was soaked in the specified film solution for 30 min, drained, and placed on a medium with a diameter of 90 mm inoculated with *Escherichia coli* ATCC 8099 and *Staphylococcus aureus* ATCC 6538 with a concentration of 1 × 10^6^ CFU/mL adjusted using a McFarland scale turbidimetric tube, respectively. The samples were incubated at 37 °C for 24 h for observation.

#### 2.3.10. Antioxidant Activity

Antioxidant activity was determined according to the method by Lin et al. [43], with minor modifications. Briefly, the film sample (3 × 3 cm) was placed in 10 mL of DPPH solution (0.01 mM) for 0.5 h in the dark. The absorbance was measured at 517 nm, and the DPPH radical scavenging rate of the sample was calculated as shown in Equation (9) as follows:

(9) $$\mathrm{DPPH}\;\mathrm{radical}\;\mathrm{scavenging}\;\mathrm{rate}\;(\%)=\frac{A_{0}-{\;A}_{1}}{A_{0}}\;\times \;100\%$$

where *A*_0_ is the absorbance at 517 nm of the blank control and *A*_1_ is the absorbance at 517 nm of the sample.

#### 2.3.11. Stability of Sesamol in C/S-ses

Sesamol stability was measured as described by Huang et al. [44], with minor modifications. The film was irradiated at a distance of 20 cm from the UV lamp (8 W, Philips, Amsterdam, The Netherlands) with a wavelength of 253.7 nm for 34 h. Sesamol was extracted by ethanol and ultrasound and measured at 431 nm. The absorbance of free sesamol was used as a control under the same conditions, and each experiment was repeated three times.

#### 2.3.12. Slow Release of Sesamol in C/S-ses

The release behavior of sesamol was measured according to the method by Huang et al. [28], with slight modifications. The film sample was immersed in PBS solution (0.01 M) with different pH values (5.2, 7.0, and 8.4) for 21 d. A solution sample (5 mL) was taken to measure the release of sesamol at 426 nm every 3 d. Seven replicates of each sample were prepared for the estimation of the sesamol release rate in film.

### 2.4. Statistical Analysis

Each set of experiments was performed three times in parallel. Experimental data were presented as mean values ± standard deviation (SD). Graphs were made in GraphPad Prism 9.4.1 (San Diego, CA, USA), and statistical analyses were performed by SPSS software 26.0 and Excel 2010 (New York, USA; Chicago, IL, USA). *p* < 0.05 indicated that there was a significant difference between the two groups.

## 3. Results and Discussion

### 3.1. Accelerated Oxidative Experiment of Walnut Kernels

#### 3.1.1. PV

**Figure 1:**
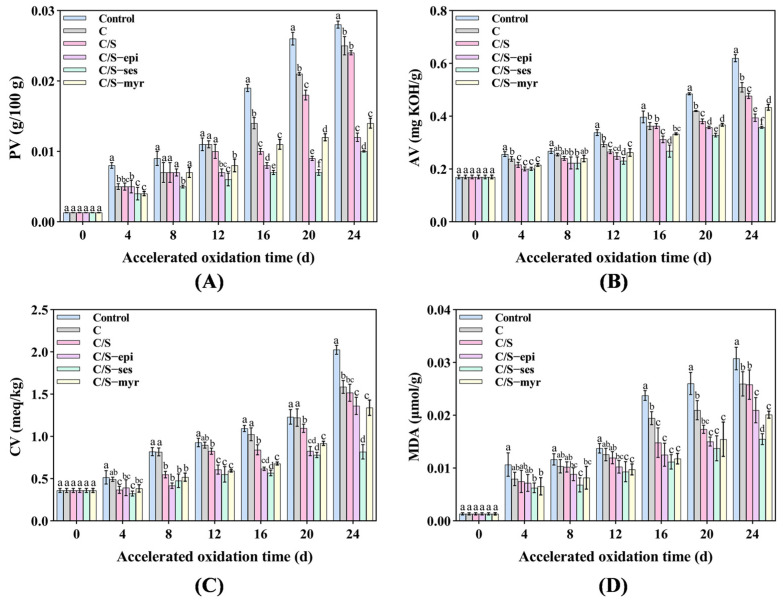
The effect of the accelerated oxidation process on PV (**A**), AV (**B**), CV (**C**), and MDA (**D**) evaluated for the walnut kernels, as control, C, C/S, C/S-epi, C/S-ses, and C/S-myr. Different letters indicate significant differences between groups (*p* < 0.05).

The PV can detect the amount of hydroperoxides produced during lipid oxidation, which increases with the degree of oxidation. All the tested samples showed the trend that the PV was gradually raised from 0 d to 24 d (Figure 1A). On day 24, the rank order of PV from most to least was as follows: Control > C > C/S > C/S-myr > C/S-epi > C/S-ses. Thus, composite films containing antioxidants effectively inhibited lipid peroxidation in walnut kernels. C/S-ses reduced the PV by 64% compared to the control group and was more effective than C/S-myr and C/S-epi (*p* < 0.05). It can be attributed to that the antioxidant film was slowly released onto the surface of walnuts to trap the active lipid radicals and prevent the oxidation chain reaction [45]. Additionally, the film covering may have inhibited oxidation by forming a barrier to UV light and ambient oxygen [46].

#### 3.1.2. AV

The AV is an indicator of free fatty acid (FFA) content and the degree of oil hydrolysis. As shown in Figure 1B, the AV increased progressively in all groups but was lower in the antioxidant film groups compared to the control, C, and C/S groups by day 24 (*p* < 0.05). Moreover, C/S-ses proved more effective for delaying the increase in AV (*p* < 0.05), which was consistent with the results of PV. The high AV in control was attributable to the presence of water and degradation of alkyl esters, while the low values in the C/S-myr, C/S-epi, and C/S-ses groups reflected the increase in oxidative activation energy (Ea) conferred by the active molecule, which hindered FFA hydrolysis [47].

#### 3.1.3. CV

The CV reflects the concentration of carbonyl compounds in the oils [48]. Figure 1C showed that the CV of walnut oil increased gradually from 0 d to 24 d. On day 24, the CV of C, C/S, C/S-epi, C/S-myr, and C/S-ses were significantly lower compared to the control group by 21%, 25%, 32%, 33%, and 59%, respectively (*p* < 0.05). Further, among the three natural antioxidants, sesamol had the best antioxidant effect on walnut preservation (*p* < 0.05), followed by myricetin and epicatechin. This was due to the fact that antioxidants can inhibit the formation of carbonyl compounds by trapping the adducts formed by carbonyl compounds [49].

#### 3.1.4. MDA

MDA reflects the degree of membrane lipid peroxidation [50]. With increasing storage time, the MDA of walnut kernels gradually increased in all groups (Figure 1D). Notably, MDA at day 24 was significantly lower in the C/S-ses group than in the C/S-myr and C/S-epi groups (*p* < 0.05), indicating that sesamol conferred higher resistance to the generation of lipid oxidation products [51]. Additionally, films containing myricetin and epicatechin also effectively inhibited the increase in MDA.

#### 3.1.5. K_232_ and K_268_

**Figure 2:**
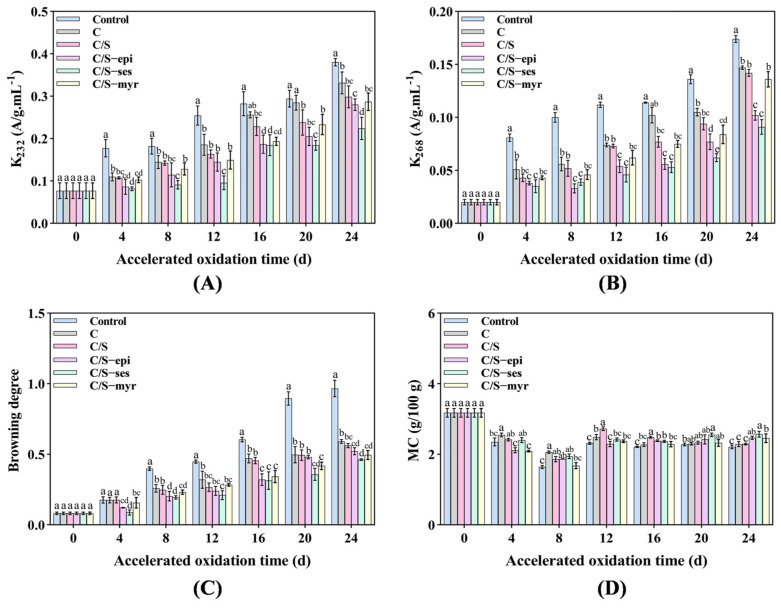
The effect of the accelerated oxidation process on K_232_ (**A**), K_268_ (**B**), browning degree (**C**), and MC (**D**) evaluated for the walnut kernels, as control, C, C/S, C/S-epi, C/S-ses, and C/S-myr. Different letters indicate significant differences between groups (*p* < 0.05).

Oxidative deterioration of oils can be detected by measuring the content of conjugated dienes and conjugated trienes, which show strong absorbance at 232 nm and 268 nm [52]. K_232_ and K_268_ are in turn related to the formation of major primary and secondary oxidation products, respectively [53]. In all samples, K_232_ was larger than K_268_ (Figure 2A,B) because the synthesis rate of primary oxidation products was much faster than that of decomposition, consistent with the change in PV [54]. On day 24, the K_232_ of C/S-ses group walnuts was reduced by 41%, that of C/S-epi and C/S-myr samples by 26% and 23%, respectively, compared to the untreated control group. Again, sesamol demonstrated the strongest antioxidant effect (*p* < 0.05). Additionally, C/S-ses yielded the lowest K_268_ among all groups, with a final value of 47% lower than that of the control group (*p* < 0.05). Values were also numerically reduced in the C/S-epi and C/S-myr groups.

#### 3.1.6. Browning Degree

The inner skin of walnut kernels is susceptible to browning under the influence of the external environment. As expected, the degree of browning increased with storage time, reaching 0.966 in the control group by day 24, which was significantly higher than all other groups (*p* < 0.05) (Figure 2C). The degree of browning was significantly lower in the C and C/S groups (*p* < 0.05) but lower still in the C/S-ses group (*p* < 0.05). The activity of polyphenol oxidase was found to be the main cause of browning, and antioxidants can effectively inhibit this activity [52,55]. Consistent with the current results [56], it was found that sesamol can effectively reduce the browning of apples, bananas, and potatoes (up to 60–65%).

#### 3.1.7. MC

MC has a great influence on walnut quality, and free water is required for almost all metabolic processes. The MC of all groups first decreased and then increased during the storage period, although the general trend was a reduction compared with before storage (Figure 2D). On day 24, the MC was lowest in the control group, indicating that film treatments enhanced walnut kernel quality in part by reducing water dissipation. Among them, the MC of walnuts in the antioxidant group was better maintained, probably related to the dense film surface.

According to these results, the developed antioxidant composite films were superior to composite films without antioxidants (and no treatment) for the preservation of walnut kernels. As sesamol exhibited the best antioxidant properties, the C/S-ses film was characterized in greater detail compared with C and C/S films.

### 3.2. Physicochemical Characterization of C, C/S, and C/S-ses Films

#### 3.2.1. Mechanical Properties and Thickness

**Figure 3:**
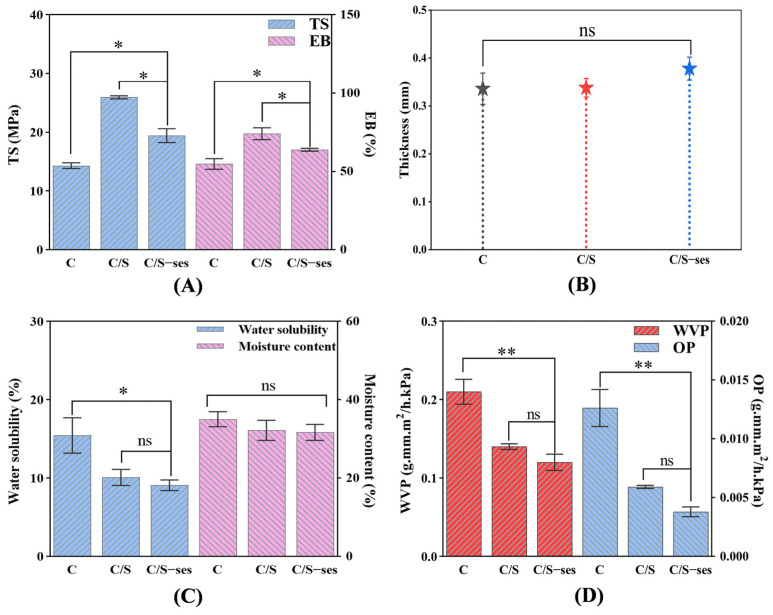
Mechanical properties (**A**), thickness (**B**), moisture content and water solubility (**C**), and WVP and OP (**D**) of C, C/S, and C/S-ses films. * indicates *p* < 0.05, ** indicates *p* < 0.01, and ns denotes non-significance.

The addition of soy protein peptide increased the TS of C film from 14.33 ± 0.28 MPa to 25.95 ± 0.27 MPa (*p* < 0.05) and the EB from 54.84 ± 3.40% to 74.11 ± 3.82% (*p* < 0.05) (Figure 3A). The improved mechanical properties of C/S film may be attributed to intermolecular hydrogen bonding between the two substances, which would increase film rigidity and durability. Additionally, the mobility of the molecular chains in C/S film likely contributed to the improved flexibility compared to C film [57,58]. It is worth noting that the addition of sesamol reduced the TS of C/S film from 25.95 ± 0.27 MPa to 19.44 ± 1.18 MPa, which could be the reason for the aggregation of the remaining sesamol on the film surface to further enhance antioxidant efficacy [59]. However, the overall performance of the C/S-ses film was greater than that of the C film (*p* < 0.05), indicating that the C/S-ses film had better applicability. The addition of soy protein peptide and sesamol also increased film thickness (Figure 3B), consistent with the related literature showing that the thickness of polysaccharide film was increased by the addition of solid components [60].

#### 3.2.2. Moisture Content and Water Solubility

Applied film for food preservation should also have good water resistance to prevent excessive moisture absorption, which can result in mold [60]. The moisture content and water solubility of the films are shown in Figure 3C. Moisture content was higher in the C film (35.03 ± 1.93%) compared to the C/S and C/S-ses films due to the greater hydrophilicity of chitosan. The water solubility of C/S and C/S-ses films decreased by 34.7% and 41.2% compared with C films, likely due to the reduction in water binding sites on chitosan when interacting with soy protein peptide and sesamol [61].

#### 3.2.3. WVP and OP

The barrier properties of composite films could be characterized using WVP and OP [62]. As can be seen from Figure 3D, C/S-ses film (0.12 ± 0.010 g·mm·m^2^/h·kPa) and C/S (0.14 ± 0.003 g·mm·m^2^/h·kPa) films (*p* > 0.05) have lower WVP compared to C film (0.21 ± 0.016 g·mm·m^2^/h·kPa) (*p* < 0.01). Additionally, the OP of C/S-ses (0.0038 ± 0.0001 g·mm·m^2^/h·kPa) and C/S films (0.0059 ± 0.0001 g·mm·m^2^/h·kPa) (*p* > 0.05) is lower than that of C film (0.013 ± 0.0016 g·mm·m^2^/h·kPa) (*p* < 0.01), which is similar to the results of WVP. A lower WVP and OP implied that the addition of sesamol and soy protein peptide resulted in a single film with a more compact molecular structure due to interactions with the hydrophilic groups of chitosan [63], thereby forming a stronger barrier to water vapor and oxygen.

#### 3.2.4. Appearance and Color

Film color can affect the appearance of food products. Table 1 shows the color parameters of the three films. With the addition of soy protein peptide and sesamol, the Δ*E* and b* increased and the a* and L* decreased, indicating a tendency for the film to turn yellow and darker. Deeper color could reduce exposure of food to visible and UV light, effectively delaying light-dependent spoilage [64].

#### 3.2.5. Opacity

The opacity of composite film affected the sensory attributes and light transmission properties of foods [65]. The rank order of film opacity was C/S-ses film > C/S film > C film (*p* < 0.05), (Table 1), likely due in part to the greater film thickness conferred by the addition of solid components and the reason for sesamol self-coloring. The high opacity of C/S-ses film suggested a greater potential for blocking light-induced oxidation reactions in fats and oils [66], which was consistent with the results for film color.

#### 3.2.6. SEM

**Figure 4:**
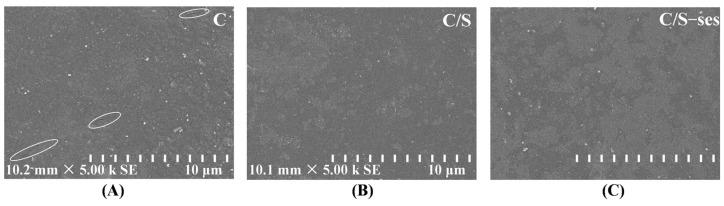
SEM micrographs acquired using 5000× magnification for the surface of the chitosan-based films for C (**A**), C/S (**B**), and C/S-ses (**C**).

The microstructure of the film surface was observed by SEM. As can be seen in Figure 4, the surface of the C film was rough and cracked, possibly due to the precipitation of dissolved chitosan particles [67]. In contrast, the C/S film exhibited a relatively smooth and dense surface structure, indicating good chemical compatibility with chitosan [68]. A few particles appeared on the C/S film surface after the addition of sesamol, but the overall structure was still tight and free of cracks, in accordance with the findings by [28]. This result also further confirmed the successful loading of sesamol into the hydrogen bonding network structure of C/S film.

#### 3.2.7. FTIR Spectroscopy

**Figure 5:**
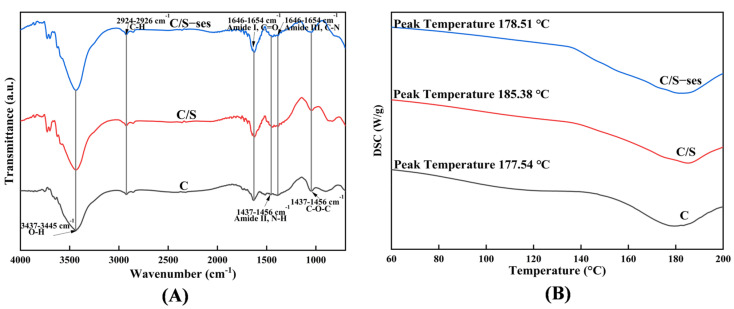
FTIR (**A**) spectra and DSC curves (**B**) recorded for C, C/S, and C/S-ses films.

FTIR spectroscopy was performed to examine the interactions among components of the composite film (Figure 5A). In the film samples, absorption peaks were detected at 3437–3445 cm^−1^ and 2924–2926 cm^−1^, which reflect O–H and C–H bond stretching, respectively. With the addition of soy protein peptide and sesamol, the peak at 3445.688 cm^−1^ in C film shifted to 3437.942 cm^−1^ and 3437.492 cm^−1^, respectively, confirming the hydrogen bonding interactions between substances [69]. These three films had characteristic absorption peaks at 1646.482–1654.142 cm^−1^ (amide I, C = O stretching), 1437.672–1456.672 cm^−1^ (amide II, N–H stretching), and 1385.603–1389.460 cm^−1^ (amide Ⅲ, C–N stretching), respectively, which suggested intermolecular interactions to form amide groups [40]. The peak at 1048.604–1060.175 cm^−1^ was attributed to the C–O–C of glycerol [70]. It can be seen that no additional absorption peaks were observed in C film after the addition of soy protein peptide and sesamol, demonstrating that these additions did not fundamentally disrupt the polymer structure.

#### 3.2.8. DSC

In this study, DSC was conducted to evaluate the thermal stability of the film. The C, C/S, and C/S-ses films exhibited heat absorption peaks at 177.54 °C, 185.38 °C, and 178.51 °C, respectively (Figure 5B). The heat flow curve peak of a polymer is usually at or near the polymer glass transition temperature (Tg), at which the material transforms from a hard or glassy state to a softer rubbery state due to the disruption of the polymer structure [71]. A higher Tg indicates greater thermal stability of the original structure and molecular chains, as more energy was required to break the molecular bonds [72]. C/S film was more difficult to melt than C film because the addition of soy protein peptide increased the intermolecular interaction forces. Notably, the addition of sesamol decreased the Tg of C/S film, although it was still higher than that of C film, which could be attributed to the reason that the excess sesamol may hinder the cross-linking between chitosan and soy protein peptide molecules, leading to a decrease in the thermal stability [73]. These changes in thermal stability resemble those in mechanical properties. Nonetheless, the enhanced antioxidant activity compensated for the reduced thermal and mechanical stability induced by sesamol.

#### 3.2.9. Antibacterial Activity

**Figure 6:**
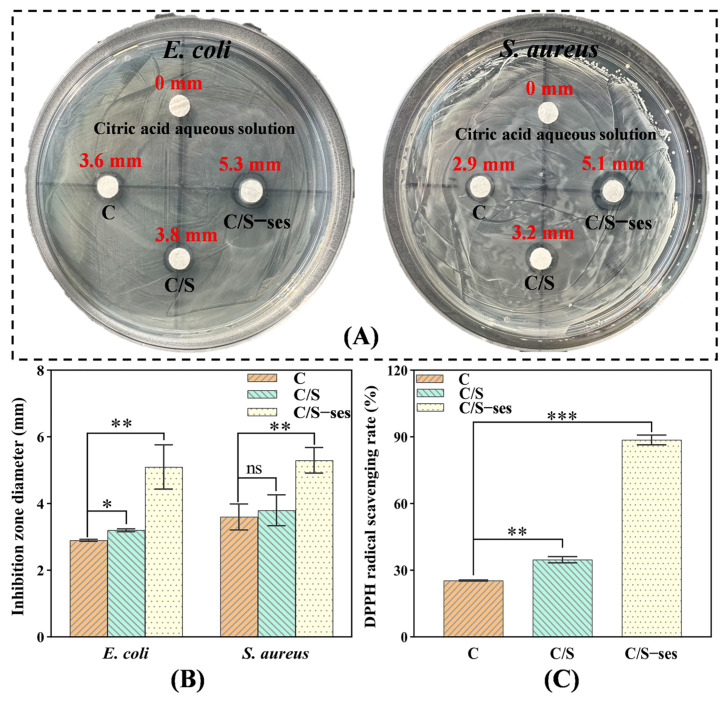
Inhibition zone diameters (**A**,**B**) and DPPH radical scavenging rates (**C**) of C, C/S, and C/S-ses films. * indicates *p* < 0.05, ** indicates *p* < 0.01, *** indicates *p* < 0.001, and ns denotes non-significance.

Recently, as recognized dangerous foodborne disease pathogens, Gram-negative *E. coli* and Gram-positive *S. aureus* have attracted much attention [45]. In this study, *E. coli* and *S. aureus* were selected as model organisms to evaluate the antimicrobial activity of different films using the zone of inhibition method. As shown in Figure 6A,B, the inhibition zone diameters of C/S-ses film on *E. coli* and *S. aureus* cultures were significantly larger (5.1 mm and 5.3 mm, respectively) than those of C film (2.9 mm and 3.6 mm, respectively) and C/S film (3.2 mm and 3.8 mm) (*p* < 0.01). Obviously, the addition of sesamol enhanced the antimicrobial properties of the polymer film. It might be attributed to the fact that sesamol damaged the bacterial wall membrane, leading to an increase in cell membrane permeability, which prevented the bacteria from maintaining normal bacterial morphology and affected the replication of DNA [60]. These results are consistent with the reported inhibitory effects of sesamol on the growth of microbial pathogens [74].

#### 3.2.10. Antioxidant Activity

The antioxidant activity of C/S-ses film as measured by DPPH radical scavenging rate was up to 88.67%, which was significantly higher than that of C and C/S-ses films (*p* < 0.001) (Figure 6C). The result was expected because of the strong antioxidant properties of sesamol reported in the relevant literature [60], which also further reflected that the antioxidant composite film was effective in inhibiting the oxidative reactions of walnut kernel lipids.

#### 3.2.11. Stability of Sesamol in C/S-ses

**Figure 7:**
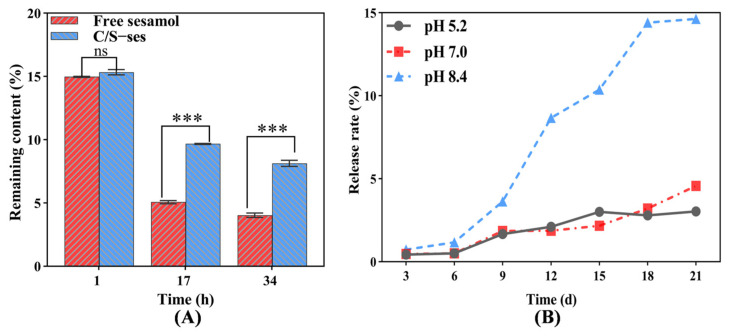
Stability of sesamol in C/S-ses (**A**) and slow release of sesamol in C/S-ses (**B**). *** indicates *p* < 0.001, and ns denotes non-significance.

To study the stability of sesamol in composite film, C/S-ses film was subjected to UV light for 32 h compared to untreated sesamol (free sesamol) under the same conditions. As can be seen in Figure 7A, there was no significant difference between free sesamol in solution and C/S-ses film after 1 h of UV exposure. However, the sesamol content was markedly reduced in both groups but was higher in C/S-ses film than in free solution at 17 h (*p* < 0.001). After 34 h of irradiation, the sesamol content in the film increased by 50.4% compared to the solution, indicating that the composite film could better maintain the stability of sesamol and allow it to exert antioxidant activity. Similar results were found by Huang et al. [28].

#### 3.2.12. Slow Release of Sesamol in C/S-ses

Slow release is an effective strategy to improve the efficient utilization of embedded substances [75]. To measure the behavior of sesamol release from composite film, C/S-ses film was incubated in different buffered solutions, and the sesamol content in solution was measured spectrophotometrically. The sesamol release rate increased gradually over time and was highest at pH 8.4 and higher at pH 7.0 than pH 5.2 at 21 d (Figure 7B). The higher release rate in alkaline solution may be attributed to disruption of the network space between chitosan and soy protein peptide, as sesamol retention depends on the network structure and solubility of the film.

## 4. Conclusions

In this study, in order to screen antioxidant films suitable for walnut kernel preservation, the different composite films based on chitosan and soy protein peptide were prepared by adding three different natural antioxidants (myricetin, epicatechin, and sesamol). The results concluded that C/S-ses provided the best protection against lipid oxidation and moisture loss compared to C/S-myr and C/S-epi. Additionally, the C/S-ses film provided superior antimicrobial and antioxidant activities compared to C and C/S films and demonstrated good mechanical as well as thermal stability. The encapsulation of the composite film improved the stability and release of sesamol, which further enhanced the antioxidant efficiency. This C/S-ses film may be a safe and effective treatment for preventing lipid oxidation and microbial contamination of walnut kernels during storage.

## Figures and Tables

**Table 1:** Appearance, color, and opacity of C film, C/S film, and C/S-ses films.

Sample	Appearance	L*	a*	b*	Δ*E*	Opacity
C	* 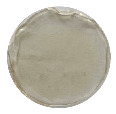 *	39.52 ±0.34 ^a^	3.49 ± 0.81 ^a^	5.37 ± 0.33 ^c^	61.02 ± 0.33 ^c^	0.27 ± 0.03 ^c^
C/S	* 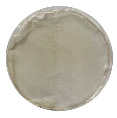 *	36.04 ± 0.72 ^b^	−4.70 ± 0.43 ^b^	7.388 ± 0.27 ^b^	64.19 ± 0.72 ^b^	0.34 ± 0.01 ^b^
C/S-ses	* 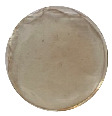 *	33.46 ± 0.67 ^c^	−4.37 ± 0.67 ^b^	8.266 ± 0.41 ^a^	67.38 ±0.58 ^a^	0.39 ± 0.02 ^a^

Different letters indicate significant differences between groups (*p* < 0.05).

## Data Availability

The original contributions presented in the study are included in the article/supplementary material, further inquiries can be directed to the corresponding author.

## Supplementary Materials

The following supporting information can be downloaded at: https://www.mdpi.com/article/10.3390/foods13091313/s1, Figure S1: Coating of walnut kernels.
