# Supplementary figures and images for: Screening and Characterization of Antioxidant Film Applicable to Walnut Kernels from Juglans sigillata
Source: Foods. 2024 Apr 25;13(9):1313. doi: 10.3390/foods13091313 (PMC11083998; doi:10.3390/foods13091313)

## Supplementary Material

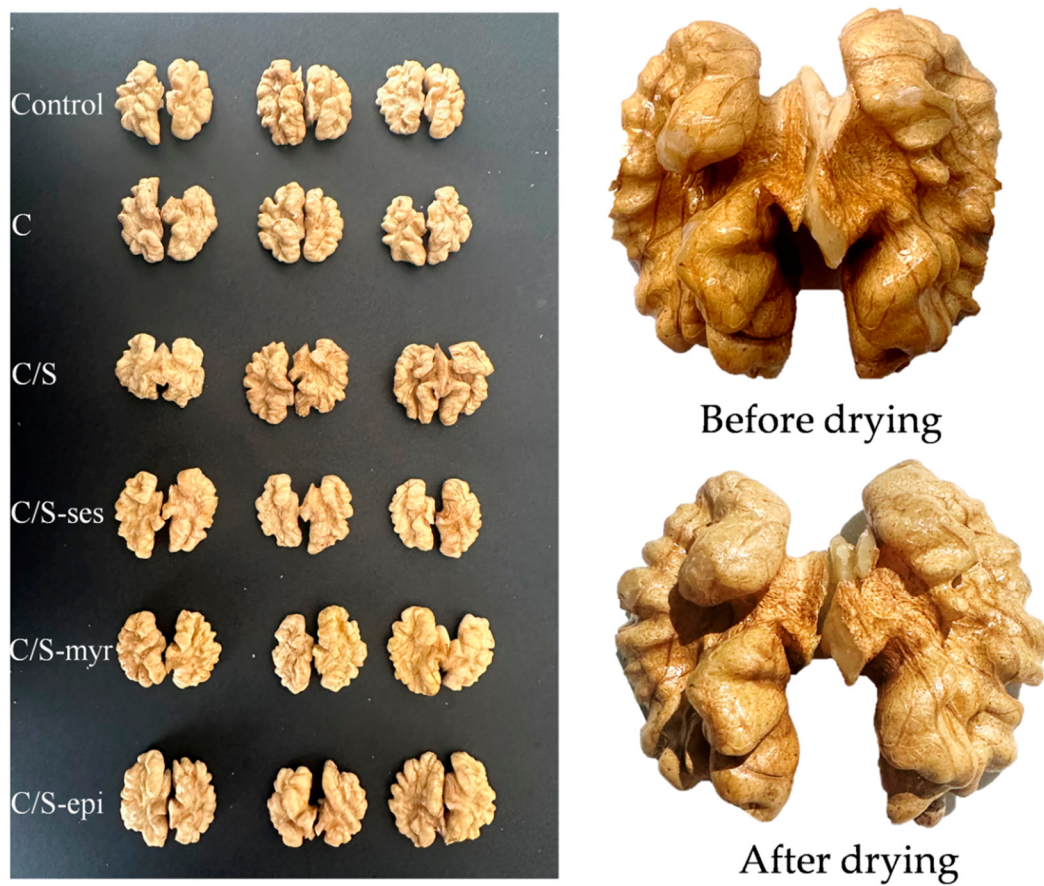

**Figure S1.** The coated walnut kernels.

Supplement: Supplementary file 1 [file foods-13-01313-s001.zip › foods-2944327-supplementary.pdf]
